# Dual tasking impairments are associated with striatal pathology in Huntington’s disease

**DOI:** 10.1002/acn3.51142

**Published:** 2020-08-14

**Authors:** Johnny Lo, Alvaro Reyes, Timothy S. Pulverenti, Timothy J. Rankin, Danielle M. Bartlett, Pauline Zaenker, Grant Rowe, Kirk Feindel, Govinda Poudel, Nellie Georgiou‐Karistianis, Mel R. Ziman, Travis M. Cruickshank

**Affiliations:** ^1^ School of Science Edith Cowan University Joondalup Western Australia Australia; ^2^ Facultad de Ciencias de la Rehabilitacion Universidad Andres Bello Viña del Mar Chile; ^3^ Department of Physical Therapy College of Staten Island The City University of New York Staten Island NY; ^4^ School of Medical and Health Sciences Edith Cowan University Joondalup Western Australia Australia; ^5^ Centre for Sleep Science School of Human Sciences Faculty of Science University of Western Australia Crawley Western Australia Australia; ^6^ School of Biomedical Sciences University of Western Australia Perth Western Australia Australia; ^7^ Mary MacKillop Institute for Health Research Australian Catholic University Melbourne Australia; ^8^ School of Psychological Sciences and the Turner Institute for Brain and Mental Health Monash University Melbourne Australia; ^9^ School of Biomedical Science University of Western Australia Crawley Western Australia Australia; ^10^ Exercise Medicine Research Institute School of Medical and Health Sciences Edith Cowan University Joondalup Western Australia Australia; ^11^ Perron Institute for Neurological and Translational Science Perth Western Australia Australia

## Abstract

**Background:**

Recent findings suggest that individuals with Huntington’s disease (HD) have an impaired capacity to execute cognitive and motor tasks simultaneously, or dual task, which gradually worsens as the disease advances. The onset and neuropathological changes mediating impairments in dual tasking in individuals with HD are unclear. The reliability of dual tasking assessments for individuals with HD is also unclear.

**Objectives:**

To evaluate differences in dual tasking performance between individuals with HD (presymptomatic and prodromal) and matched controls, to investigate associations between striatal volume and dual tasking performance, and to determine the reliability of dual tasking assessments.

**Methods:**

Twenty individuals with HD (10 presymptomatic and 10 prodromal) and 20 healthy controls were recruited for the study. Individuals undertook four single and dual task assessments, comprising motor (postural stability or force steadiness) and cognitive (simple or complex mental arithmetic) components, with single and dual tasks performed three times each. Participants also undertook a magnetic resonance imaging assessment.

**Results:**

Compared to healthy controls, individuals with presymptomatic and prodromal HD displayed significant deficits in dual tasking, particularly cognitive task performance when concurrently undertaking motor tasks (*P* < 0.05). The observed deficits in dual tasking were associated with reduced volume in caudate and putamen structures (*P* < 0.05),however, not with clinical measures of disease burden. An analysis of the reliability of dual tasking assessments revealed moderate to high test–retest reliability [ICC: 0.61‐0.99] for individuals with presymptomatic and prodromal HD and healthy controls.

**Conclusions:**

Individuals with presymptomatic and prodromal HD have significant deficits in dual tasking that are associated with striatal degeneration. Findings also indicate that dual tasking assessments are reliable in individuals presymptomatic and prodromal HD and healthy controls.

## Introduction

The simultaneous execution of cognitive and motor tasks (*i.e*., dual tasking) is essential for activities of daily living, including driving and maintaining balance.^1^, ^2^Recent evidence suggests that individuals with Huntington’s disease (HD) have greater difficulty executing dual tasks than healthy controls, which adversely impacts on their functional independence and quality of life.^3^, ^4^

Existing studies have documented deficits in bimanual tapping, walking while talking and speed accuracy trade‐off tasks in individuals with manifest HD.^3^, ^5^, ^6^, ^7^, ^8^, ^9^ Such deficits worsen with increasing task difficulty and are predictive of cognitive and motor impairments in HD.^4^, ^6^, ^10^While these findings provide compelling evidence that dual tasking impairments are a prominent feature of manifest HD, whether these deficits exist during the premanifest stages and whether progressive striatal degeneration underpins these deficits remains unclear.

The striatum, which shows early and strikingly selective degeneration in HD,^11^, ^12^, ^13^ has been suggested to be fundamentally involved in dual tasking.^14^ Recent studies in individuals with Parkinson’s disease (PD) noted significant associations between greater striatal damage and dual tasking deficits.^15^, ^16^To our knowledge, associations between striatal degeneration and dual tasking have not been investigated in individuals with HD. Considering the early loss of functional segregation within the striatum,^17^, ^18^it is likely that striatal degeneration underlies, at least in part, dual tasking deficits observed in individuals with HD. Studies are nevertheless needed to test this supposition. There is also a fundamental need to evaluate whether dual tasking deficits arise during the premanifest stages of HD. During this period of the disease, participants are characterized as having presymptomatic HD(pre‐HD; little to no clinical signs) orprodromal HD (pro‐HD; subtle, but unequivocal signs to warrant formal diagnosis of the disease). Treatment therapies are likely to have greatest therapeutic effect during this period of the disease, when most neural structures are intact and remain remediable to therapeutic strategies. It is, therefore, of vital importance to have sensitive measures of disease onset and progression to enable effective trailing of novel therapeutic strategies.

This study aimed to: (1) characterize differences in dual tasking between individuals with pre‐HD and pro‐HD and healthy age‐ and gender‐matched controls, (2) investigate associations between striatal volume and dual tasking performance, and (3) determine the reliability of dual task assessments in individuals with HD. Based on existing evidence, we hypothesized that individuals with HD would exhibit significant deficits in dual tasking, compared to healthy controls, and that such deficits would be associated with greater striatal degeneration.

## Methods and Materials

### Ethical approval and patient consent

All research procedures were conducted in accordance with the Declaration of Helsinki. Ethical approval for this study was granted by Edith Cowan University (13145) and North Metropolitan Area Mental Health Service (2009‐16) Human Research Ethics Committees. Researchers ensured that all participants understood the requirements of the study. Written and informed consent was provided by all participants.

### Participants

Twenty individuals with premanifest HD(10 pre‐HD and 10 pro‐HD) and 20 healthy age‐and gender‐matched controls were recruited for this study. Inclusion criteria for individuals with pre‐HD and pro‐HD were as follows: a CAG repeat length >39, a diagnostic confidence level score of ≤2 and a total functional capacity (TFC) score of 13 ( of a possible 13, indicating highest functional capacity) on the Unified Huntington’s Disease Rating Scale [UHDRS‐TMS].^19^Individuals with a UHDRS‐TMS score ≥ 5, a diagnostic confidence level (DCL) score of 2 and cognitive impairments (as indicated by a composite score comprising: Hopkins Verbal Learning Test, Symbol Digit Modalities Test, Trail Making Test Part A and B, Cambridge One Touch Stockings) were classified as pro‐HD and individuals with a UHDRS‐TMS score < 5, a DCL score between 0 and 2 and no validated clinical signs were classified as pre‐HD.^20^ Individuals with pre‐HD and pro‐HD were excluded from the study if they had recent or ongoing substance abuse or concomitant musculoskeletal, cardiovascular, or sleep disorders. Inclusion and exclusion criteria for healthy age‐ and gender‐matched controls were as follows: no family history of HD, no recent or ongoing substance abuse and no known neurological, musculoskeletal, cardiovascular, or sleep disorders. Disease burden score (DBS) is a measure of genetic burden and was calculated using the method described by Penney et al. (1997).^21^ An index to estimate proximity to diagnosis at study entry was obtained using the CAP score (CAG‐Age Product Scaled score). CAP score was calculated by multiplying the age at study entry by a scaling of the CAG repeat length as follows: CAP_S_ = (Age × (CAG‐33.66))/432.3326. CAP scores <1, 1 and >1 indicate a 5‐year diagnosis probability of <0.5, 0.5, and >0.5, respectively.^22^

### Experimental design

Similar to recent work in individuals with PD,^15^ participants were asked to perform cognitive and motor tasks simultaneously to examine dual tasking ability. Each task was performed for 20 seconds. To ensure the reliability of single and dual task assessments: (a) the same examiners were used to administer assessments, b) participants were familiarized with single and dual task assessments prior to administration, (c) assessments were administered in the same order for participants (cognitive tasks prior to motor tasks and single tasks prior to dual tasks [see Table 3 and 4 for testing order]), and (d) test–retest data were collected to evaluate the reliability of assessments for individuals with pre‐HD, pro‐HD and healthy controls. Importantly, all assessments, including clinical (UHDRS‐TMS), dual tasking, and neuroimaging assessments were performed within 4 weeks for all participants. All examiners had significant experience working with people with HD and therefore were not blinded to group status. Single and dual task assessments were performed three times each. Specific information on cognitive and motor tasks are detailed below.

#### Cognitive tasks

Two numeracy assessments were used to evaluate cognitive performance under single and dual task conditions. Numeracy assessments included the Serial Threes Test (STT) and a Progressive Subtraction Test (PST).^23^ The STT requires participants to make multiple subtractions of three from a whole three‐digit number, *e.g*., 256, 253, 250, 247. The PST required participants to verbalize progressive subtractions of 1, 2, 3, 4, and 5 from a three‐digit whole number and the resulting minuends, e.g., 455, 453, 450, and 446. Different three‐digit numbers were used for each trial to reduce learning effects. Numeracy tests were purposefully selected to evaluate cognitive performance given their proven sensitivity in individuals with HD and ecological validity (use in everyday life, particularly the management of finances and time).^24^

#### Motor tasks

Postural stability and force steadiness tasks were used to evaluate motor performance under single and dual task conditions. The sensory organization test (SOT) on the Neurocom Smart Balance Master was used to evaluate postural stability. The SOT comprises six different sensory conditions: 1) eyes open, fixed support, and surroundings, 2) eyes closed, fixed support, and surroundings, 3) eyes open, fixed support, moving surroundings, 4) eyes open, unstable support, fixed surroundings, 5) eyes closed, unstable support, fixed surroundings, and 6) eyes open, unstable support, and moving surroundings. For the purpose of this study, only eyes open conditions (1, 3, 4, and 6) were used to ensure that outcomes were ecologically relevant. Values are expressed as a percentage of the theoretical maximum angle of sway, therefore a score of 100 indicates good stability and no movement of the centre of gravity. The Biodex System 4 was used to evaluate force steadiness in the right plantar flexors at 10% of the maximum voluntary force that participants can generate. Prior to the commencement of force steadiness trials participants performed three maximum voluntary isometric contractions (MVIC) of the right plantar flexors. The highest force (Nm) generated in a single MVIC trial was recognized as the participant’s maximum force output and was used to calculate a 10% submaximal plantar flexion contraction target force. With a television screen positioned in front of the participant, the participant was asked to maintain the real‐time force generation line on the horizontal target force line (10% MVIC). The amplitude of force fluctuations above and below the horizontal target force line (force steadiness) during trials was quantified and used for analysis. The higher the force fluctuation the worse the performance. These tests were selected based on their proven sensitivity in individuals with HD.^25^, ^26^, ^27^, ^28^, ^29^, ^30^For more detailed information see Supplementary files.

#### Dual task performance

Dual task cost (DTC) values were calculated and analysed to assess performance on dual tasks. The formula used to calculate dual task cost values is provided below^31^

$$\text{DTC}\left(\%\right)=‐\left(\left(\text{dual task performance}‐\text{single task performance}\right)/\left(\text{single task performance}\right)\right)\times 100.$$

This formula enables calculation of DTC values (% change) for cognitive and motor components of each dual task. Negative values indicate a reduction in cognitive and motor performance during dual tasking, compared to single task performance, indicating an interference effect or dual task cost.

#### MRI acquisition and analysis

T_1_‐weighted structural images of the brain were obtained from each participant using a GE Healthcare Discovery MR750W 3T MRI scanner. Images were acquired with a 24‐channel head coil using a 3DIR‐SPGR sequence (TA = 9 m 59 s, TR = 3 s, TE = 3.1 ms, TI = 400 ms, flip angle = 11˚, field of view = 256 mm^3^, image matrix = 256 × 256 × 256, 1 mm^3^ isotropic voxels). The T_1_‐weighted MRI images were automatically processed with the processing pipeline available in FreeSurfer.^32^ FreeSurfer was used to parcellate the T_1_‐weighted MRI data into cortical and subcortical brain regions according to the Desikan–Killiany atlas. The analyses were performed on MASSIVE HPC (www.massive.org.au) using the “recon‐all” function. The neuroanatomical labels were inspected for accuracy in all HD and healthy control cases. Using the FreeSurfer processing outputs (aseg.stats), we extracted volume of the striatum (caudate and putamen), which were used in statistical analyses.

#### Statistical analysis and estimated sample size

Sample size was calculated based on the results reported by Vaportzis et al^5^, ^6^ on dual task cost in individuals with manifest HD and controls performing two level tasks. At the time of this study, there were no previous studies describing dual task cost in individuals with pre‐HD or pro‐HD. The sample size calculation was therefore based on three group effects and interactions. From the results of these previous studies it suffices that the minimum detectable effect size (Eta‐squared) is set at 0.07. For a mixed‐model analysis of variance (ANOVA), to examine the effects of group, gender, task, and interactions and using an alpha level of 0.05, a statistical power of 0.8, it was estimated a sample size of at least 10 participants per group.

Reliability of the single and dual tasks for each group are given by the intraclass correlation (ICC), estimated with a two‐way mixed model for absolute agreement. These values were estimated using trials collected as part of the single dual tasking testing session. For ICC, values less than 0.5, between 0.5 and 0.75, between 0.75 and 0.9, and greater than 0.90 are indicative of poor, moderate, good, and excellent reliability respectively.^33^

Mixed‐model analysis of variance (ANOVA) was used to examine the effects of task (single vs. dual), group (pre‐HD, pro‐HD vs. control), gender (male vs. female) and the two‐way task × group and task × gender interactions on STT, PST, force steadiness, and postural stability, whilst adjusting for participant’s age. Pairwise comparisons were conducted with Bonferroni post‐hoc test, and where appropriate, contrasts were used to compare the premanifest and prodromal HD individuals collectively to healthy controls.

Effect sizes for the ANOVAs are described by partial Eta‐squared with 0.01, 0.06, and 0.14 identified as small, medium, and large effects respectively. Post‐hoc effect sizes are described by Cohen’s *d* with small, medium, and large effect sizes (in absolute terms) defined by 0.2, 0.5, and 0.8 respectively.^34^

General linear modeling (GLM) was used to assess the associations between dual task performance with clinical disease outcomes and striatal volume separately for pro‐HD, pre‐HD, and the healthy controls. The GLMs for the HD groups were adjusted for gender and CAP as covariates, whilst gender and age were accounted for in healthy control GLMs. Benjamini‐Hochberg correction was applied to all *p*‐values in order to minimize false discovery rate. Within group analysis for dual task performance and dual task cost are presented in the Supplementary Files. All analyses were carried out using IBM SPSS version 25 (IBM SPSS, Chicago, IL). Results were considered significant at *P* ≤ 0.05.

## Results

### Demographic characteristics

Participants completed all experimental procedures required for the study. Clinical and demographic data are presented in Table 1. No significant differences were observed between groups (pro‐HD, pre‐HD and control) for gender. However, a significant difference in age was observed between groups (*P* = 0.038), whereby the pro‐HD subjects were significantly older than the pre‐HD (*P* = 0.035). A significant difference was also observed for the cognitive composite score, with individuals with pro‐HD displaying significantly reduced performance compared to healthy controls.

**Table 1:** Clinical and demographic characteristics of study cohorts.

Variable	Pre‐HD (*n* = 10)	Pro‐HD (*n* = 10)	Control (*n* = 20)	*P* value
Gender; n (%)				0.270^a^
Male	5 (50%)	2 (20%)	5 (25%)	
Female	5 (50%)	8 (80%)	15 (75%)	
Age^1^	36.5 (8.6)	50.1 (14.1)	42.1 (11.3)	0.038^b^
CAG repeats^2^	43.0 (42.0, 44.0)	41.5 (40.0, 44.2)	‐	
DBS^1^	297.5 (84.6)	313.4 (86.2)	‐	
CAP score^1^	0.8 (0.2)	0.9 (0.2)	‐	
UHDRS‐TMS^2^	0.5 (0.0, 1.2)	10.0 (7.8, 21.2)	‐	
DCL^2^	0.0 (0.0, 0.0)	1.0 (0.8, 2.0)	‐	
TFC	13 (0)	13 (0)	‐	
Cognitive composite score	0.007 (2.93)	−1.44 (2.52)	1.27 (1.07)	0.004^c^

Pre‐HD: premanifest individuals with HD, Pro‐HD: prodromal individuals with HD, CAG: cytosine‐adenine‐guanosine, DBS: disease burden score, CAP score: CAG‐Age Product Scaled score, UHDRS‐TMS: Unified Huntington´s Disease Rating Scale‐Total Motor Score, DCL: diagnostic confidence level, TFC: total functional capacity score of the UHDRS‐TMS.

^1^ Normally distributed data, mean (SD) are presented.

^2^ Data are non‐normal, median (*Q*_1_, *Q*_3_) are presented.

^a^ Chi‐square test.

^b^ Independent *t*‐test

^c^ Kruskal–Wallis test (control vs. pre‐HD, *P* = 0.111; control vs. pro‐HD *P* = 0.002; pre‐HD vs. pro‐HD, *P* = 0.081).

### Test–retest reliability of single and dual task tests

Single task and dual task test–retest data are presented in Table 2. Single task and dual task STT, PST, and force steadiness assessments demonstrated moderate to excellent test–retest reliability, with intraclass correlation coefficient (ICC) values ranging between 0.61and 0.99. Single task and dual task postural stability outcomes demonstrated slightly lower test–retest reliability, with ICC values ranging from 0.50 to 0.94.

**Table 2:** Reliability of single‐task and dual‐task outcomes (intraclass correlation coefficients).

Single Task	Dual Task	Pre‐HD	Pro‐HD	Control
Serial threes test		0.99	0.99	0.98
	Force Steadiness	0.99	0.99	0.99
	Postural stability			
	Condition 1	0.92	0.80	0.96
	Condition 3	0.94	0.95	0.96
	Condition 4	0.96	0.93	0.97
	Condition 6	0.90	0.93	0.96
Progressive subtraction test		0.92	0.92	0.95
	Force Steadiness	0.98	0.98	0.98
	Postural stability			
	Condition 1	0.82	0.88	0.83
	Condition 3	0.87	0.92	0.61
	Condition 4	0.97	0.93	0.88
	Condition 6	0.97	0.86	0.78
Force Steadiness		0.95	0.94	0.84
	Serial threes test	0.91	0.72	0.84
	Progressive subtraction test	0.97	0.65	0.89
Postural stability				
Condition 1		0.56	0.73	0.85
Condition 3		0.73	0.65	0.59
Condition 4		0.79	0.83	0.93
Condition 6		0.71	0.94	0.73
	Serial threes test			
Condition 1		0.50	0.72	0.83
Condition 3		0.90	0.90	0.91
Condition 4		0.91	0.84	0.84
Condition 6		0.87	0.94	0.86
	Progressive subtraction test			
Condition 1		0.88	0.82	0.83
Condition 3		0.84	0.88	0.82
Condition 4		0.94	0.90	0.94
Condition 6		0.74	0.93	0.87

### Group differences in single and dual task‐performance

Tables 3 and 4 outline the significant variables/interactions identified in mixed models for single and dual tasks and dual task costs respectively. The post‐hoc results are as follows. There were no significant differences in single and dual tasks performance and dual task costs between pre‐HD and pro‐HD.

**Table 3:** Single and dual task performance for pre‐HD, pro‐HD, and healthy controls using mixed‐model ANOVAs. Models were adjusted for gender and age. Adjusted means (95% confidence intervals) are presented for each group. Only the notable interactions/variables (*P* < 0.05) are shown in the table.

Single Task	Dual Task	Pre‐HD	Pro‐HD	Control	Notable Interaction/Variable	*P* value	Partial Eta‐Squared
Serial threes test		11.6 (8.9, 14.3)	10.2 (7.4, 12.9)	10.9 (9.0, 12.8)	task × group	<0.001	0.429
	Force steadiness^a^, ^b^	9.1 (5.9, 12.3)	8.5 (5.2, 11.7)	15.8 (13.6, 18.0)			
	Postural stability						
	Condition 1	9.8 (7.3, 12.2)	7.9 (5.4, 10.4)	11.0 (9.3, 12.7)			
	Condition 3^b^	10.3 (8.1, 12.6)	8.1 (5.8, 10.4)	11.4 (9.8, 13.0)			
	Condition 4	10.6 (8, 13.2)	8.2 (5.6, 10.9)	11.5 (9.7, 13.4)			
	Condition 6^b^	11.4 (8.8, 13.9)	7.7 (5.2, 10.3)	12.3 (10.5, 14.0)			
Progressive subtraction test		6.6 (5.4, 7.8)	6.1 (4.9, 7.4)	7.5 (6.6, 8.3)	task × group	<0.001	0.221
	Force steadiness^a^, ^b^	5.6 (4.0, 7.2)	5.1 (3.5, 6.7)	8.1 (7.0, 9.2)			
	Postural stability						
	Condition 1	7.2 (6.1, 8.3)	6.5 (5.3, 7.6)	6.5 (5.7, 7.3)			
	Condition 3	6.8 (5.8, 7.8)	6.3 (5.3, 7.3)	7.7 (7.0, 8.4)			
	Condition 4	6.2 (4.9, 7.4)	5.6 (4.2, 6.9)	6.8 (5.9, 7.7)			
	Condition 6^a^, ^b^	6.1 (4.9, 7.2)	5.7 (4.6, 6.8)	8.1 (7.3, 8.9)			
Force steadiness^c^		3.0 (1.7, 4.4)	3.3 (1.8, 4.8)	2.1 (1.1, 3.1)	group	0.031	0.180
	Serial threes test^c^	5.4 (3.0, 7.8)	4.9 (2.4, 7.5)	2.3 (0.5, 4.0)			
	Progressive subtraction test^c^	4.1 (2.4, 5.9)	5.2 (3.3, 7.1)	2.3 (1.1, 3.6)			
Postural stability					stability × age	0.024	0.252
Condition 1		93.8 (92.5, 95.1)	92.8 (91.4, 94.1)	95.1 (94.2, 96.0)			
Condition 3		63.2 (55.3, 71.1)	66.1 (58.0, 74.2)	70.3 (64.8, 75.8)			
Condition 4		80.5 (73.0, 88.1)	80.3 (72.5, 88.0)	76.4 (71.1, 81.6)			
Condition 6		83.3 (79.4, 87.3)	85.3 (81.3, 89.3)	86.6 (83.8, 89.3)			
	Serial threes test						
Condition 1		90.4 (87.9, 92.9)	87.8 (85.2, 90.4)	93.3 (91.6, 95.1)			
Condition 3		89.3 (85.9, 92.6)	87.8 (84.3, 91.2)	90.9 (88.6, 93.3)			
Condition 4		60.4 (50.4, 70.3)	55.7 (45.5, 65.8)	65.3 (58.4, 72.3)			
Condition 6		76.7 (69.9, 83.4)	77.8 (70.9, 84.7)	78.3 (73.6, 83.0)			
	Progressive subtraction test						
Condition 1		87.0 (81.0, 93.0)	88.8 (82.7, 95.0)	84.5 (80.3, 88.7)			
Condition 3		86.5 (83.0, 90.0)	86.6 (83.0, 90.2)	89.6 (87.2, 92.0)			
Condition 4		85.7 (80.8, 90.7)	89.5 (84.4, 94.5)	87.2 (83.7, 90.6)			
Condition 6		61.3 (52.1, 70.5)	56.5 (47.1, 65.9)	65.3 (58.9, 71.7)			

^a^ Significant difference between Pre‐HD and Control (*P* < 0.05).

^b^ Significant difference between Pro‐HD and Control (*P* < 0.05).

^c^ Significant difference between Pre‐HD and Pro‐HD (*P* < 0.05).

**Table 4:** Dual task cost results for pre‐HD, pro‐HD, and healthy controls using mixed‐model ANOVAs. Models were adjusted for gender and age. Adjusted means (95% confidence intervals) are presented for each group. Only the notable interactions/variables (*P* < 0.05) are shown in the table.

Dual Task Costs (%)		Pre‐HD	Pro‐HD	Control	Notable Interaction/Variable	*P*‐value	Partial Eta‐Squared
Serial threes test	Force steadiness^a^, ^b^	−19.5 (−36.2, −2.7)	−20.8 (−37.9, −3.7)	48.5 (36.8, 60.2)	task × group	<0.001	0.417
	Postural stability						
	Condition 1^b^	−9.9 (−23.6, 3.8)	−21.1 (−35.1, −7.1)	1.7 (−7.9, 11.3)			
	Condition 3^b^	−4.3 (−18.5, 9.9)	−18.7 (−33.2, −4.2)	6.8 (−3.1, 16.8)			
	Condition 4^b^	−4.4 (−18.7, 9.9)	−16.9 (−31.5, −2.3)	7.3 (−2.7, 17.3)			
	Condition 6^b^	3.1 (−11.2, 17.4)	−18.5 (−33.1, −3.8)	13.5 (3.5, 23.5)			
Progressive subtraction test	Force steadiness^b^, ^c^	−12.8 (−29.7, 4.2)	−21.2 (−38.6, −3.9)	10 (−1.9, 21.9)	task × group	<0.001	0.257
	Postural stability						
	Condition 1^c^	14.7 (−4.2, 33.6)	14.3 (−5.1, 33.6)	−12.5 (−25.7, 0.7)			
	Condition 3	4.0 (−6.5, 14.5)	5.6 (−5.2, 16.3)	4.0 (−3.3, 11.3)			
	Condition 4	−8.9 (−21.2, 3.5)	−10.5 (−23.1, 2.2)	−8.1 (−16.7, 0.5)			
	Condition 6^c^	−10.6 (−25.6, 4.5)	−6.3 (−21.6, 9.1)	10.1 (−0.4, 20.6)			
Force steadiness	Serial threes test^b^	−109.4 (−185.3, −33.6)	−37.1 (−118.7, 44.6)	−7.5 (−63.1, 48.0)	group	0.042	0.165
	Progressive subtraction test^b^	−56.8 (−104.7, −8.9)	−57.2 (−108.8, −5.6)	−15.5 (−50.6, 19.6)			
Postural stability^1^	Serial threes test				age	0.040	0.118
Condition 1		−4.8 (−8.5, −1.1)	−5.4 (−9.2, −1.6)	−4.4 (−7.0, −1.8)			
Condition 3		−7.8 (−14.2, −1.4)	−9.8 (−16.3, −3.2)	−9.7 (−14.1, −5.2)			
Condition 4		−8.6 (−13.5, −3.7)	−3.6 (−8.6, 1.5)	−8.4 (−11.8, −4.9)			
Condition 6		−12.3 (−26.0, 1.4)	−13.1 (−27.1, 0.9)	−5.5 (−15.0, 4.1)			
	Progressive subtraction test						
Condition 1		−4.5 (−7.6, −1.3)	−1.3 (−4.5, 1.9)	−4.0 (−6.2, −1.8)			
Condition 3		−4.3 (−23.1, 14.5)	−23.7 (−42.9, −4.4)	−4.5 (−17.6, 8.7)			
Condition 4		−7.8 (−11.2, −4.5)	−3.0 (−6.4, 0.5)	−7.3 (−9.6, −4.9)			
Condition 6		−2.1 (−17.9, 13.7)	−21.6 (−37.8, −5.4)	−5.4 (−16.5, 5.6)			

^1^ Outcome is inversely associated with age.

^a^ Significant difference between Pre‐HD and Control (*P* < 0.05).

^b^ Significant difference between Pro‐HD and Control (*P* < 0.05).

^c^ Significant difference between (Pre‐HD + Pro‐HD) and Control (*P* < 0.05) through contrast.

#### Single task performance

Performance on SOT, force steadiness, PST and STT tasks did not differ significantly between groups (*P* > 0.05). Age was negatively associated with performance on PST (*P* = 0.002) and postural stability tasks (*P* < 0.024).

### Dual task performance

Overall, individuals with pre‐HD and pro‐HD, compared with controls, made fewer correct subtractions on the STT task when concurrently undertaking the force steadiness task (pre‐HD + pro‐HD *P* = 0.001; large ES = −1.219; pre‐HD *P* = 0.003; *d *= −1.435 and pro‐HD *P* = 0.001; *d* = −1.556). Furthermore, pro‐HD individuals performed better than the controls on the STT task when concurrently undertaking condition 4 (*P* = 0.046, *d* = 0.987) and condition 6 (*P* = 0.011, *d* = 1.209) of the SOT task. Similarly, pre‐HD and pro‐HD individuals made significantly fewer correct subtractions on the PST task than healthy controls when concurrently undertaking the force steadiness task (HD *P* = 0.008; *d *= −1.022, pre‐HD *P* = 0.041; *d* =−1.043 and pro‐HD *P* = 0.008; *d* =−1.254), and as well as condition 6 (HD *P < *0.001; *d *= −1.623, pre‐HD *P* = 0.011; *d *= −1.250 and pro‐HD *P* = 0.002; *d* = −1.452) of the SOT task. Pre‐HD and pro‐HD individuals performed significantly worse on the force steadiness task when concurrently undertaking STT or PST tasks than healthy controls (HD *P* = 0.009, *d *= −0.886). Age was negatively associated (*P* = 0.024) with postural stability during single task and when concurrently undertaking STT and PST (dual task).

### Dual task cost analyses

**Figure 1:**
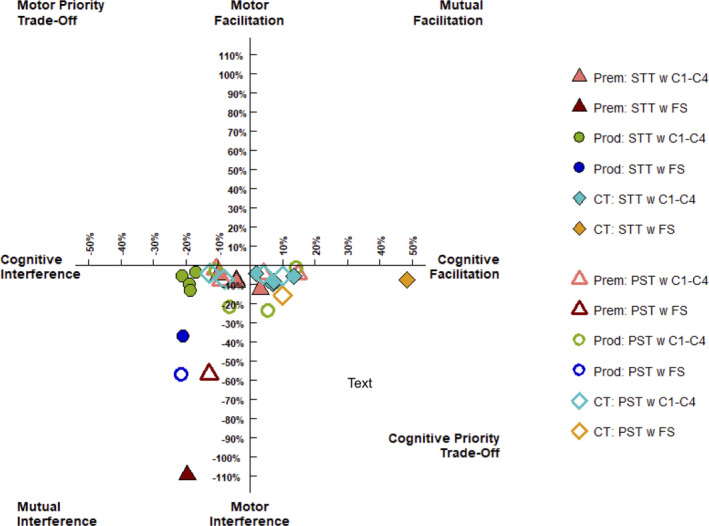
Patterns of dual task cost according to the Plummer and Eskes framework.^31^ Prem: premanifest HD, Prod: prodromal HD, CT: controls, STT: serial threes test, PST: progressive subtraction test, FS: force steadiness, C1‐C4: conditions 1 to 4 of the sensory organisation test.

Compared with healthy controls, individuals with HD (pre‐HD and pro‐HD) demonstrated a deteriorated performance on the STT task when concurrently undertaking the force steadiness task (HD *P* < 0.001, *d *= −2.457; pre‐HD *p* < 0.001, *d *= −2.746 and pro‐HD *P* < 0.001, *d *= −2.778). The pre‐HD and pro‐HD groups overall also demonstrated poorer performance than healthy controls on the PST when concurrently undertaking force steadiness task (HD *P* = 0.001, *d *= −1.112). Compared with healthy controls, individuals with pro‐HD demonstrated a deteriorated performance on the STT task when concurrently undertaking condition 1 (*P* = 0.002, *d *= −1.118), condition 3 (*P* = 0.011; *d *= −1.029), condition 4 (*P* = 0.017; *d *= −1.140), and condition 6 (*P* = 0.001; *d *= −1.498) of the SOT task, but no significant differences were observed with pre‐HD. The pre‐HD and pro‐HD groups overall also demonstrated poorer performance than healthy controls on the PST when concurrently undertaking condition 6 of the SOT task (HD *P* = 0.011, *d *= −0.0.861). Contrary with expectations, pre‐HD and pro‐HD individuals overall exhibited better performance on the PST than healthy controls when concurrently undertaking condition 1 of the SOT task (HD *P* = 0.004, *d* = 0.996). Individuals with pre‐HD performed more poorly in the force steadiness task when concurrently undertaking either STT or PST than the healthy controls (HD *P* = 0.048, *d *= −1.018). No interference effects were found for postural stability tasks when undertaking cognitive tasks. However, age was negatively associated with postural stability across all SOT conditions (*P* = 0.040). Figure 1 provides a visual representation of the patterns of dual task cost for individuals with pre‐HD, pro‐HD, and healthy controls. The figure shows that individuals with pre‐HD and pro‐HD demonstrated reduced cognitive performance when performing the SOT and the force steadiness tasks.

Given the small sample size and lack of significant differences observed between pre‐HD and pro‐HD subgroups, associations among dual task performance, clinical disease outcomes, and striatal volume, were undertaken with both pre‐HD and pro‐HD groups amalgamated.

#### Associations between dual task performance and clinical disease outcomes in individuals with HD

No consistent associations were found between dual task outcomes and clinical disease outcomes. STT DTC values when undertaking the force steadiness task were negatively associated with DBS (*coefficient:*‐0.11, 95% CI −0.17 to −0.04, *P* = 0.014) and CAP (*coefficient:*‐46.19, 95% CI −74.73 to −17.95, *P* = 0.014). A negative association was also found between UHDRS‐TMS and condition 6 of the postural stability task when undertaking the STT task (*coefficient:*‐2.26, 95% CI −3.75 to −0.78, *P* = 0.046).

#### Associations between dual task performance and striatal volume

Significant associations were observed between caudate and putamen volume and performance on the STT when HD participants (pre‐HD and pro‐HD) were concurrently undertaking the force steadiness tasks (left caudate; *coefficient:*4.92, 95% CI 1.76 to 8.07, *P* = 0.034; right caudate; *coefficient:*6.69, 95% CI 3.64 to 9.74, *P* = 0.006; left putamen; *coefficient:*3.04, 95% CI 2.04 to 4.04, *P* = 0.001; right putamen,*coefficient:*2.88, 95% CI 0.86 to 4.91, *P* = 0.038). Similar associations were found for the PST and force steadiness dual task and caudate and putamen volume in individuals with HD (pre‐HD and post‐HD) (left caudate; *coefficient:*3.21, 95% CI 1.59 to 4.82, *P* = 0.007; right caudate; *coefficient:*3.64, 95% CI 1.81 to 5.47,* P* = 0.007; left putamen; *coefficient:*1.44, 95% CI 0.69 to 2.19, *P* = 0.007). There were no significant associations between caudate and putamen volume and performance on force steadiness and postural stability tasks when undertaking subtraction tasks (*P *> 0.05). There were no associations between caudate and putamen volume and dual task outcomes in healthy controls (*P *> 0.05).

## Discussion

We investigated differences in dual tasking between individuals with pre‐HD, pro‐HD, and healthy controls, and for the first time, associations between striatal volume and dual tasking performance. In addition, we also investigated the reliability of dual tasking assessments in individuals with pre‐HD, pro‐HD, and healthy controls. Our study revealed three main findings. First, individuals with pre‐HD and pro‐HD, when compared to healthy controls, display significant deficits in dual tasking, particularly cognitive task performance when concurrently undertaking motor tasks. Second, task‐specific deficits in dual tasking are associated with striatal degeneration, but not clinical measures of disease burden. Third, the assessed dual tasking assessments show acceptable test–retest reliability in individuals with pre‐HD, pro‐HD, and healthy controls.

### Dual tasking performance

Consistent with previous studies in manifest HD,^4^, ^5^, ^6^, ^7^, ^9^, ^10^, ^35^, ^36^ we found significant task‐specific deficits in dual tasking in individuals with pre‐HD and pro‐HD. Performance appeared to be moderated by age, with older individuals and females displaying greater deficits in dual tasking. Compared to healthy controls, individuals with pre‐HD and pro‐HD exhibited a significant deterioration in cognitive performance (fewer correct subtractions) when concurrently undertaking motor tasks (force steadiness or postural stability tasks). Interestingly, interference effects appeared to be greater for the simple arithmetic task (STT), when concurrently undertaking force steadiness and postural stability tasks. This finding was unexpected. We expected greater interference effects for the complex arithmetic task (PST).While speculative, it is possible that the cognitive tasks used in the present study engaged different cognitive abilities that are differentially affected in HD.^37^ In particular, the STT under dual tasking conditions may be more reliant on information processing abilities, whereas the PST may be more reliant on problem solving and memory abilities, which are less impacted in the premanifest stages of HD.^37^Future studies are needed to evaluate the clinical factors influencing dual tasking impairments in HD.

While not significant, our results also show that cognitive tasks have a marked interference effect on motor tasks under dual task conditions, which aligns with previous studies that have documented an interference effect of cognitive tasks on walking and balance under dual task conditions.^3^, ^4^According to the task prioritization model proposed by Yogev‐Seligmann et al. (2012),^38^individuals prioritize specific motor or cognitive tasks to avoid hazardous situations while under dual task conditions. Based on this model, it is conceivable that individuals with HD prioritized postural stability tasks rather than arithmetic tasks to avoid falling, however, this requires further investigation.^31^Together, these findings indicate mutual interference of cognitive and motor tasks under dual task conditions, however, more pronounced interference effects for cognitive rather than motor task performance individuals with pre‐HD and pro‐HD.

### Neuropathological associations with dual tasking performance

We found that deficits in dual tasking were more pronounced in individuals with greater degeneration in caudate and putamen structures in individuals with HD (pre‐HD and pro‐HD). To our knowledge, this is the first study to report associations between striatal degeneration and dual tasking in individuals with HD (pre‐HD and pro‐HD). This finding was not surprising given the early degeneration of the striatum in HD and the role of the striatum in the parallel processing of cognitive and motor activities^39^ and prioritization of tasks under dual tasking conditions.^31^Interestingly, our findings are in alignment with those reported in individuals with PD, who also exhibit striatal pathology. Nieuwhof et al (2017) documented significant associations between pathological alterations in striatal activation and dual tasking deficits in individuals with PD. While tentative, these results suggest that striatal degeneration, at least in part, underpins deficits in dual tasking in individuals with HD (pre‐HD and pro‐HD) and perhaps individuals with striatal pathology in general. These cross‐sectional findings nevertheless need to be confirmed in larger observational studies.

### Associations of clinical measures of disease burden and dual tasking performance

Contrary with our expectations, no consistent associations were observed between measures of disease burden and dual tasking performance in individuals with HD (pre‐HD and pro‐HD). These results are not overly surprising and are likely attributed to the insensitivity of disease burden measures during the premanifest stages of the disease, particularly considering the observed associations between striatal damage and dual tasking performance.

### Study limitations

This study is not without limitations. First, this was a cross‐sectional study, which did not enable examination of the sensitivity of these measures over time. Second, this study included individuals with pre‐HD and pro‐HD, findings are therefore not generalizable across the spectrum of the disease. Additional studies are needed to determine whether the examined dual tasks are sensitive over time and across the spectrum of the disease. Third, this study only examined associations between striatal damage and dual task performance and therefore do not reflect a causal link between striatal pathology and dual tasking deficits. Future interventional studies designed to interfere with striatal activity, for example noninvasive brain stimulation techniques or pharmacological therapies, are needed to determine a causal link between striatal damage and dual task deterioration. Finally, we only assessed associations between caudate and putamen brain structures and dual tasking performance, as these are the principle brain structures affected by the disease and sensitive to early disease changes. It is important to note that other brain structures are also affected, including the cerebellum and brain stem,^40^, ^41^ which may also influence dual tasking in HD.^42^, ^43^ Additional studies should investigate the role of other subcortical and cortical structures and dual tasking.

## Conclusions

Our findings show, for the first time, that dual tasking impairments are present in individuals with pre‐HD pro‐ HD and are associated with striatal degeneration. Furthermore, our findings show that dual tasking measures are reliable in individuals with pre‐HD and pro‐HD. These findings are of clinical interest given the negative impact of dual tasking impairments on activities of daily living. Furthermore, these findings are of interest given the urgent need for sensitive measures of disease burden for upcoming disease modifying drug trials. Finally, these findings are of clinical interest to rehabilitation specialists, who could use the examined dual tasking paradigms to identify individuals with dual tasking impairments that may benefit from dual task training, which has demonstrated efficacy in individuals with PD, who similarly display striatal damage and associated dual tasking deficits. Larger longitudinal studies are nevertheless needed to confirm these findings.

## Conflict of Interest

The authors declare no competing interests.

## Supporting information


**Figure S1.** Schematic representation of the Sensory Organization Test (SOT).The SOT comprises six different sensory conditions: 1) eyes open, fixed support, and surroundings (static posturography), 2) eyes closed, fixed support, and surroundings, 3) eyes open, fixed support, moving surroundings, 4) eyes open, unstable support, fixed surroundings, 5) eyes closed, unstable support, fixed surroundings, and 6) eyes open, unstable support and moving surroundings. Individuals were required to undertake three 20‐second trials for each sensory condition. For each trial, participants were instructed to stand upright with their arms crossed against their chest. Postural stability performance on each trial was expressed as an equilibrium score, which is calculated by computing the difference between each participant´s sway of the centre of gravity (COG) and a theoretical maximum anterior‐posterior sway of 12.5º. When a participant´s COG has minimal or no sway, the difference with the theoretical maximum sway is 12.5º. Values are expressed as a percentage of the theoretical maximum angle of sway, therefore, a score of 100 indicates good stability and no movement of the COG. When a participant’s COG moves beyond the limit of stability or the participant has a fall, they receive a score of zero.

**Supplementary Material.** Force Steadiness testing details and sensory organization test calculation. Results of the within group analysis for dual task performance and dual task cost.
