# Supplementary figures and images for: Dual tasking impairments are associated with striatal pathology in Huntington’s disease
Source: Ann Clin Transl Neurol. 2020 Aug 14;7(9):1608–19. doi: 10.1002/acn3.51142 (PMC7480913; doi:10.1002/acn3.51142)

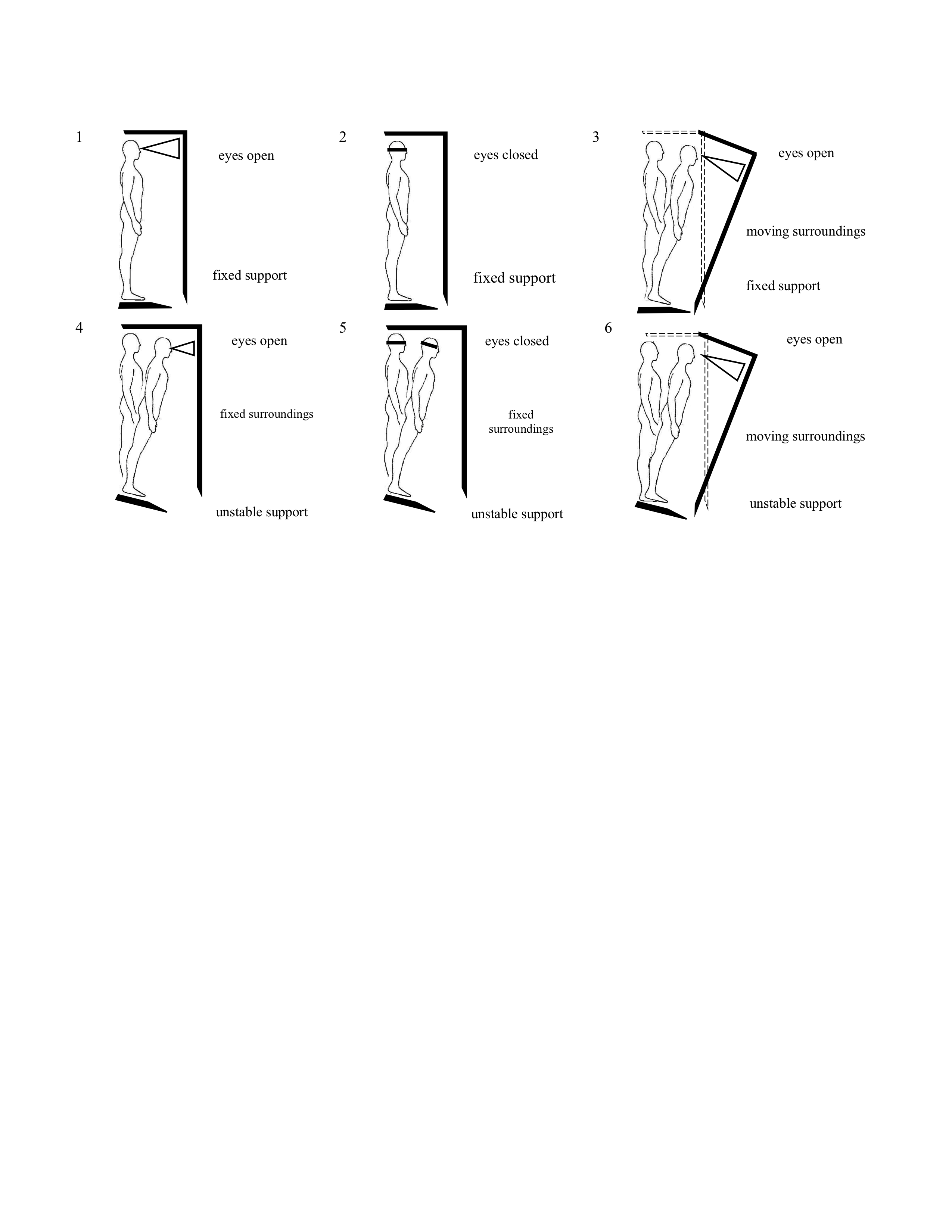

Supplement: Supplementary file 1 — Figure S1. Schematic representation of the Sensory Organization Test (SOT).The SOT comprises six different sensory conditions: 1) eyes open, fixed support, and surroundings (static posturography), 2) eyes closed, fixed support, and surroundings, 3) eyes open, fixed support, moving surroundings, 4) eyes open, unstable support, fixed surroundings, 5) eyes closed, unstable support, fixed surroundings, and 6) eyes open, unstable support and moving surroundings. Individuals were required to undertake three 20‐second trials for each sensory condition. For each trial, participants were instructed to stand upright with their arms crossed against their chest. Postural stability performance on each trial was expressed as an equilibrium score, which is calculated by computing the difference between each participant´s sway of the centre of gravity (COG) and a theoretical maximum anterior‐posterior sway of 12.5º. When a participant´s COG has minimal or no sway, the difference with the theoretical maximum sway is 12.5º. Values are expressed as a percentage of the theoretical maximum angle of sway, therefore, a score of 100 indicates good stability and no movement of the COG. When a participant’s COG moves beyond the limit of stability or the participant has a fall, they receive a score of zero. [file ACN3-7-1608-s001.tiff]
