# Supplementary material for: Dual tasking impairments are associated with striatal pathology in Huntington’s disease
Source: Ann Clin Transl Neurol. 2020 Aug 14;7(9):1608–19. doi: 10.1002/acn3.51142 (PMC7480913; doi:10.1002/acn3.51142)
Supplement: Supplementary file 2 — Supplementary Material. Force Steadiness testing details and sensory organization test calculation. Results of the within group analysis for dual task performance and dual task cost. [file ACN3-7-1608-s002.docx]

**Supplementary Material**

**Set-Up for Force Steadiness Testing**

Before commencing force steadiness testing, the participant performed a standardised warm-up on a Monark cycle ergometer consisting of five minutes of stationary cycling at 60 rpm with a 1-kg resistance. When subsequently seated on the dynamometer, the participant was positioned so that the right knee was fully extended (0°), the ankle was in a neutral position (0°; plane of foot relative to tibia), the sole of the foot was perpendicular to the shank and the lateral malleolus of the fibula aligned with the dynamometer’s centre of rotation. The participant’s upper body, knee and ankle were firmly secured to the dynamometer with straps and the contralateral foot rested on a support to avoid unwanted movements. Once secured in position, the participant performed a specific warm up consisting of four, three second isometric plantar flexion trials ranging from 20 – 80% of perceived maximum effort with 30 seconds of rest between contractions. Data collection was undertaken thereafter. All data was recorded synchronously at 2,000 Hz on a personal computer running LabChart software (version 8.1.5, ADInstruments, NSW, Australia) using a 16-bit analogue-to-digital converter (PowerLab 16/35, ADInstruments).

**Sensory Organisation Test Calculation**

Postural stability performance on each trial was expressed as an equilibrium score, which is calculated by computing the difference between each participant´s sway of the centre of gravity (COG) and a theoretical maximum anterior-posterior sway of 12.5º. When a participant´s COG has minimal or no sway, the difference with the theoretical maximum sway is 12.5º. Values are expressed as a percentage of the theoretical maximum angle of sway, therefore a score of 100 indicates good stability and no movement of the COG. When a participant’s COG moves beyond the limit of stability or the participant has a fall, they receive a score of zero.

**RESULTS**

*Within Group Analyses*

**Dual Task Performance**

Within group analyses revealed that premanifest HD made significantly fewer correct subtractions on the STT task when concurrently undertaking condition 1 (*p*=0.004, large ES=0.938) and 3 (*p*=0.031, large ES=0.765) of the SOT task, compared to number of correct subtractions on the single STT task. When assessed for gender differences, females with premanifest HD demonstrated fewer correct subtractions on the PST when concurrently undertaking the force steadiness task (*p*=0.033, large ES=-0.919), compared to number of correct subtractions on the single PST task. Performance on the PST was not impaired in individuals with premanifest HD when concurrently undertaking the SOT task (*p*>0.05 across all conditions). Healthy controls made significantly more correct subtractions on the STT task when concurrently undertaking the force steadiness task (*p*<0.001, large ES=1.603), compared to number of correct subtractions on the single STT task. For the PST, healthy controls made significant fewer correct subtractions when concurrently undertaking condition 4 (*p*=0.025, large ES=-1.529) of the SOT task, compared to the number of correct subtractions on the single PST.

**Dual Task Cost Analyses**

The analysis revealed that individuals with premanifest HD demonstrated less cognitive cost (better performance) in the PST task when concurrently undertaking condition 1 and 3 of the SOT in comparison with the cognitive cost in the PST task when concurrently undertaking force steadiness and conditions 4 and 6 of the SOT (all *p*<0.039; all medium to large ES>0.710). Healthy controls had less cognitive cost on the STT when concurrently undertaking the force steadiness tasks in comparison with the cost on the STT when concurrently undertaking condition 4 of the SOT (all *p*<0.001, all large ES >1.29). Healthy controls demonstrated less cognitive cost in the PST task when concurrently undertaking condition 6 of the SOT compared with the cost in the PST task when concurrently undertaking condition 1 (*p*=0.049; medium to large ES=0.673) and 4 (*p*=0.023; medium to large ES=0.38) of the SOT.
